# The Woman Worker's World

**Published:** 1918-08-31

**Authors:** 


### THE WOMAN WORKER'S WORLD.

We have perhaps something to learn from the German care of illegitimate children. To the town of Leipzig belongs the credit of first formulating a scheme which placed all such children under the care of paid doctors and nurses. In some places the town authorities arrange with the Infant Welfare Centres for their care. Official guardianship, where instituted, solves well the difficult problem of recovering part maintenance from the father. The Berlin authority, established in 1912, had in two years taken over nearly 5,000 children, and recovered for them over £50,000.

In issuing their regulations under which grants are payable to day nurseries in England and Wales, the Board of Education state that a revision has been made so as to provide for the payment of a grant at a rate not exceeding 50 per cent. of the net expenditure after deducting payments made for the care of children and other similar receipts. The limit of 4d. for each attendance has been withdrawn. The Board express the hope that these increased contributions, which will be payable during the current financial year and based on the expenditure incurred during the twelve months ending on March 31, 1918, may prove of assistance to managers not only in meeting the increased charges due to war conditions, but also in carrying out improvements in the nurseries, which are in some cases highly desirable. It is a condition of the recognition of a day nursery under these regulations that infants and young children should ordinarily attend for not less than nine hours a day on not fewer than five days a week; and in fixing the rate of grant the Board take into consideration the scope, character, and efficiency of the work of the institution.

Dr. Truby King's second pamphlet* is of outstanding interest and value, especially in view both of the serious tooth-troubles of our soldiers and of the new comprehension of dangers to the young generation's development. If another argument for universal return to breast-feeding and pre-natal care of the mother were needed, it would be found in his description of the wonderful system of tooth-formation in the unborn babe, a myriad little cells working, one corps to build the ivory, another to enamel its surface. Equally wonderful is the story of "thirty-two little squads of cells for building the permanent teeth" which "assemble at their proper stations seven months before the baby is born," but set seriously to work at about the time of birth. Whether either of these tasks—and the second is done once for the whole life-time—is properly accomplished depends on whether rich red blood is supplied to the gums throughout the building time. This, of course, must depend first upon the healthy life of the mother, then on that of the child; and as regards this last Dr. King emphatically declares that the infant must learn at once to work for its living. The first exercise, even of a swaddled infant, is sucking, and that must be done vigorously. If a bottle must be used the hole in its teat should be as small as possible, and sometimes mother or nurse should even keep a slight pull upon the bottle to awaken energy. As soon as possible the baby should gnaw something hard, to promote blood circulation and the flow of saliva. The wonderful teeth and fine formation of breathing spaces and jaws among Maoris who have not become debased by imitating European customs are shown in illustrations which contrast these painfully with some depraved modern conditions among our own people.

* *The Story of the Teeth, and how to save them.* By F. Truby King, C.M.G., M.B. (Whitcomb and Tombs. 1s. net.)

Dr. Truby King's explanation of absorption of roots of temporary teeth, like the sap of leaves in autumn and the tails of tadpoles, and of the damage done to a child's mouth by the early loss of molars throwing the whole set out of balance, and of the manifold dangers of decayed teeth should convince mothers of the need for dentist's supervision throughout childhood. While careful cleansing of the teeth from the time that some half-dozen of the first set are in evidence is recommended, the practice of wiping out the sucking infant's mouth is as strongly deprecated as one possible cause of thrush and adenoids. The early chewing of hard-food, biscuit, rusk, or bone, and raw apple as the Maoris masticate fern-root is highly praised, but chocolates do not find favour. The booklet of thirty-two pages costs a shilling. It would be well if it could be published—in perhaps a slightly popularised form—at 2d. or 3d. for wide circulation among working-class parents and others, as a valuable contribution to literature that promotes national health.

